# Supplementary material for: Pressures in the Ivory Tower: An Empirical Study of Burnout Scores among Nursing Faculty
Source: Int J Environ Res Public Health. 2023 Mar 1;20(5):4398. doi: 10.3390/ijerph20054398 (PMC10002003; doi:10.3390/ijerph20054398)
Supplement: Supplementary file 1 [file ijerph-20-04398-s001.zip › ijerph-2234396-supplementary.pdf]

SUPPLEMENTARY DOCUMENT: Showing table containing all analysis including those significant and those not significant.

Note: Factors highlighted in yellow shows significant differences in burnout scores or subscale across categories

a. **Kruskal Wallis test to determine if total burnout scores (BO), exhaustion (EXH), cynicism (CYN), and professional efficacy (PEFIC) subscale of Maslach Burnout Inventory General Survey (MBI-GS) differs in rank categories**

Education (3 categories):

BO

Education	Obs	Rank sum
Bachelors/Other	85	27461
Masters	340	110284
PhD	220	70590
chi2(2) = 0.047	chi2(2) with ties = 0.047	
Prob = 0.9767	Prob = 0.9767	

EXH

Education	Obs	Rank sum
Bachelors/Other	85	28467.5
Masters	340	110106.5
PhD	220	69761
chi2(2) = 0.575	chi2(2) with ties = 0.577	
Prob = 0.7501	Prob = 0.7495	

CYN

Education	Obs	Rank sum
Bachelors/Other	85	26469.5
Masters	340	110277
PhD	220	71588.5
chi2(2) = 0.383	chi2(2) with ties = 0.384	
Prob = 0.8256	Prob = 0.8253	

PEFIC

Education	Obs	Rank sum
Bachelors/Other	85	28020
Masters	340	110653
PhD	220	69662
chi2(2) = 0.423	chi2(2) with ties = 0.424	
Prob = 0.8094	Prob = 0.8089	

b. **Kruskal Wallis test to determine if total burnout scores (BO), exhaustion (EXH), cynicism (CYN), and professional efficacy (PEFIC) subscale of Maslach Burnout Inventory General Survey (MBI-GS) differs in tenure categories (5 categories and 3 categories)**

Tenure (5 categories):

BO

TenureCat	Obs	Rank sum
Clinical track	92	27628.5
Non-tenure track	92	27628.5
Teaching track	149	47194
Tenure track (research)	84	27609
Tenured	152	49655
chi2(4) = 2.345		chi2(4) with ties = 2.346
Prob = 0.6727		Prob = 0.6725

EXH

TenureCat	Obs	Rank sum
Clinical track	92	27404
Non-tenure track	149	47881.5
Teaching track	168	56210.5
Tenure track (research)	84	28116
Tenured	152	48723
chi2(4) = 2.693		chi2(4) with ties = 2.700
Prob = 0.6105		Prob = 0.6093

CYN

TenureCat	Obs	Rank sum
Clinical track	92	27988
Non-tenure track	149	46316
Teaching track	168	55796.5
Tenure track (research)	84	27269
Tenured	152	50965.5
chi2(4) = 2.640		chi2(4) with ties = 2.645
Prob = 0.6198		Prob = 0.6189

PEFIC

TenureCat	Obs	Rank sum
Clinical track	92	30195
Non-tenure track	149	48410
Teaching track	168	53728
Tenure track (research)	84	23656.5
Tenured	152	52345.5
chi2(4) = 6.279		chi2(4) with ties = 6.298
Prob = 0.1793		Prob = 0.1780

Tenure (3 categories):

BO

TenureCat	Obs	Rank sum
Clinical/Teaching Track	260	83877
Non-tenure track	149	47194
Tenure/Tenure Track	236	77264
chi2(2) = 0.300		chi2(2) with ties = 0.300
Prob = 0.8606		Prob = 0.8605

EXH

TenureCat	Obs	Rank sum
Clinical/Teaching Track	260	83614.5
Non-tenure track	149	47881.5
Tenure/Tenure Track	236	76839
chi2(2) = 0.072		chi2(2) with ties = 0.072
Prob = 0.9647		Prob = 0.9646

CYN

TenureCat	Obs	Rank sum
Clinical/Teaching Track	260	83784.5
Non-tenure track	149	46316
Tenure/Tenure Track	236	78234.5
chi2(2) = 1.129		chi2(2) with ties = 1.132
Prob = 0.5685		Prob = 0.5679

PEFIC

TenureCat	Obs	Rank sum
Clinical/Teaching Track	260	83923
Non-tenure track	149	48410
Tenure/Tenure Track	236	76002
chi2(2) = 0.022		chi2(2) with ties = 0.022
Prob = 0.9891		Prob = 0.9890

c. **Kruskal Wallis test to determine if total burnout scores (BO), exhaustion (EXH), cynicism (CYN), and professional efficacy (PEFIC) subscale of Maslach Burnout Inventory General Survey (MBI-GS) differs in rank categories (5 and 4 categories)**

Rank (5 categories):

BO

RankCat	Obs	Rank sum
Assistant Prof	144	46897
Associate Professor	230	76780.5
Clinical/Sessional Instructor	101	29429
Full Professor	88	27707
Instructor/Lecturer	82	27521.5
chi2(4) = 4.26	chi2(4) with ties = 4.262	
Prob = 0.3719	Prob = 0.3717	

EXH

RankCat	Obs	Rank sum
Assistant Prof	144	48835
Associate Professor	230	75637.5
Clinical/Sessional Instructor	101	30061.5
Full Professor	88	27380.5
Instructor/Lecturer	82	26420.5
chi2(4) = 3.535	chi2(4) with ties = 3.544	
Prob = 0.4725	Prob = 0.4712	

CYN

RankCat	Obs	Rank sum
Assistant Prof	144	45153
Associate Professor	230	76857.5
Clinical/Sessional Instructor	101	29527
Full Professor	88	28539.5
Instructor/Lecturer	82	28258
chi2(4) = 5.035	chi2(4) with ties = 5.045	
Prob = 0.2837	Prob = 0.2827	

PEFIC

RankCat	Obs	Rank sum
Assistant Prof	144	42887.5
Associate Professor	230	72661.5
Clinical/Sessional Instructor	101	33667
Full Professor	88	30151.5
Instructor/Lecturer	82	28967.5
chi2(4) = 6.41	chi2(4) with ties = 6.43	
Prob = 0.1706	Prob = 0.1693	

Rank (4 categories):

BO

RankCat	Obs	Rank sum
Assistant Prof	144	46897
Associate Professor	230	76780.5
Clinical/Sessional Instructor/Lecturer	183	56950.5
Full Professor	88	27707
chi2(3) = 1.708<br>Prob = 0.6352		chi2(3) with ties = 1.708<br>Prob = 0.6351

EXH

RankCat	Obs	Rank sum
Assistant Prof	144	48835
Associate Professor	230	75637.5
Clinical/Sessional Instructor/Lecturer	183	56482
Full Professor	88	27380.5
chi2(3) = 2.749<br>Prob = 0.4320		chi2(3) with ties = 2.756<br>Prob = 0.4308

CYN

RankCat	Obs	Rank sum
Assistant Prof	144	45153
Associate Professor	230	76857.5
Clinical/Sessional Instructor/Lecturer	183	57785
Full Professor	88	28539.5
chi2(3) = 1.475<br>Prob = 0.6880		chi2(3) with ties = 1.478<br>Prob = 0.6874

PEFIC

RankCat	Obs	Rank sum
Assistant Prof	144	42887.5
Associate Professor	230	72661.5
Clinical/Sessional Instructor/Lecturer	183	62634.5
Full Professor	88	30151.5
chi2(3) = 5.892<br>Prob = 0.1170		chi2(3) with ties = 5.91<br>Prob = 0.1160

d. **Kruskal Wallis test to determine if total burnout scores (BO), exhaustion (EXH), cynicism (CYN), and professional efficacy (PEFIC) subscale of Maslach Burnout Inventory General Survey (MBI-GS) differs in hours worked (4 categories)**

Hours Worked:

BO

HrsworkCat	Obs	Rank sum
35 hours or less	86	19586
36-40 hours	121	32563
41-45 hours	119	34617.5
45+ hours	319	121568.5
chi2(3) = 67.126		chi2(3) with ties = 67.152
Prob = 0.0001		Prob = 0.0001

EXH

HrsworkCat	Obs	Rank sum
35 hours or less	86	18842
36-40 hours	121	31763
41-45 hours	119	35833
45+ hours	319	121897
chi2(3) = 73.248		chi2(3) with ties = 73.437
Prob = 0.0001		Prob = 0.0001

CYN

HrsworkCat	Obs	Rank sum
35 hours or less	86	21275.5
36-40 hours	121	34468.5
41-45 hours	119	34061.5
45+ hours	319	118529.5
chi2(3) = 45.530		chi2(3) with ties = 45.617
Prob = 0.0001		Prob = 0.0001

PEFIC

HrsworkCat	Obs	Rank sum
35 hours or less	86	27861
36-40 hours	121	43105
41-45 hours	119	39146.5
45+ hours	319	98222.5
chi2(3) = 6.067		chi2(3) with ties = 6.086
Prob = 0.1084		Prob = 0.1075

Hours Worked (Dunn's Test):

Dunn's Pairwise Comparison of BO by HrsworkCat
(Bonferroni)

Col Mean-Row Mean	35 hours	36-40 ho	41-45 ho
36-40 ho	-1.574478<br>0.3461		
41-45 ho	-2.395304<br>0.0498	-0.905836<br>1.0000	
45+ hour	-6.774457<br>0.0000	-5.629478<br>0.0000	-4.506759<br>0.0000

Dunn's Pairwise Comparison of EXH by HrsworkCat
(Bonferroni)

Col Mean-Row Mean	35 hours	36-40 ho	41-45 ho
36-40 ho	-1.653902<br>0.2944		
41-45 ho	-3.114170<br>0.0055	-1.607134<br>0.3241	
45+ hour	-7.209993<br>0.0000	-6.020201<br>0.0000	-4.052227<br>0.0002

Dunn's Pairwise Comparison of CYN by HrsworkCat
(Bonferroni)

Col Mean-Row Mean	35 hours	36-40 ho	41-45 ho
36-40 ho	-1.427238<br>0.4605		
41-45 ho	-1.474181<br>0.4213	-0.056896<br>1.0000	
45+ hour	-5.489898<br>0.0000	-4.362147<br>0.0000	-4.267427<br>0.0001

e. **Kruskal Wallis test to determine if total burnout scores (BO), exhaustion (EXH), cynicism (CYN), and professional efficacy (PEFIC) subscale of Maslach Burnout Inventory General Survey (MBI-GS) differs in percentage of hours teaching (3 categories)**

% Hours Teaching:

BO

HRS_Teach	Obs	Rank sum
0-20%	403	124317
21-40%	179	64116
>40%	63	19902
chi2(2) = 8.922<br>Prob = 0.0115	chi2(2) with ties = 8.926<br>Prob = 0.0115	

EXH

HRS_Teach	Obs	Rank su3
0-20%	403	125021.5
21-40%	179	63264.5
>40%	63	20049
chi2(2) = 6.709<br>Prob = 0.0349	chi2(2) with ties = 6.727<br>Prob = 0.0346	

CYN

HRS_Teach	Obs	Rank sum
0-20%	403	124829
21-40%	179	63462.5
>40%	63	20043.5
chi2(2) = 7.208<br>Prob = 0.0272	chi2(2) with ties = 7.222<br>Prob = 0.027	

PEFIC

HRS_Teach	Obs	Rank sum
0-20%	403	134386.5
21-40%	179	52454
>40%	63	21494.5
chi2(2) = 6.498<br>Prob = 0.0388	chi2(2) with ties = 6.519<br>Prob = 0.0384	

% Hours Teaching (Dunn's Test):

Dunn's Pairwise Comparison of BO by HRS_TechCate
(Bonferroni)

Col Mean-Row Mean	0-20%	21-40%
21-40%	-2.970632<br>0.0045	
>40%	-0.294208<br>1.0000	1.549372<br>0.1819

Dunn's Pairwise Comparison of EXH by HRS_TechCate
(Bonferroni)

Col Mean-Row Mean	0-20%	21-40%
21-40%	-2.584717<br>0.0146	
>40%	-0.317740<br>1.0000	1.290984<br>0.2951

Dunn's Pairwise Comparison of CYN by HRS_TechCate
(Bonferroni)

Col Mean-Row Mean	0-20%	21-40%
21-40%	-2.678579<br>0.0111	
>40%	-0.333112<br>1.0000	1.334319<br>0.2731

Dunn's Pairwise Comparison of PEFIC by HRS_TechCate
(Bonferroni)

Col Mean-Row Mean	0-20%	21-40%
21-40%	2.419088<br>0.0233	
>40%	-0.306172<br>1.0000	-1.766436<br>0.1160

f. **Kruskal Wallis test to determine if total burnout scores (BO), exhaustion (EXH), cynicism (CYN), and professional efficacy (PEFIC) subscale of Maslach Burnout Inventory General Survey (MBI-GS) differs in percentage of hours research (3 categories)**

% Hours Research:

BO

**HRS_Res**	**Obs**	**Rank sum**
0-20%	564	182148.5
21-40%	61	19938.5
>40%	20	6248
chi2(2) = 0.091<br>Prob = 0.9556	chi2(2) with ties = 0.091<br>Prob = 0.9556	

EXH

**HRS_Res**	**Obs**	**Rank sum**
0-20%	564	181303
21-40%	61	20792.5
>40%	20	6239.5
chi2(2) = 0.669<br>Prob = 0.7157	chi2(2) with ties = 0.671<br>Prob = 0.7151	

CYN

**HRS_Res**	**Obs**	**Rank sum**
0-20%	564	182544
21-40%	61	19437
>40%	20	6354
chi2(2) = 0.057<br>Prob = 0.972	chi2(2) with ties = 0.057<br>Prob = 0.972	

PEFIC

**HRS_Res**	**Obs**	**Rank sum**
0-20%	564	181971
21-40%	61	19778
>40%	20	6586
chi2(2) = 0.028<br>Prob = 0.9863	chi2(2) with ties = 0.028<br>Prob = 0.9863	

g. **Kruskal Wallis test to determine if total burnout scores (BO), exhaustion (EXH), cynicism (CYN), and professional efficacy (PEFIC) subscale of Maslach Burnout Inventory General Survey (MBI-GS) differs in percentage of clinic hours (3 categories)**

% Hours Clinic:

BO

**HRS_Clinic**	**Obs**	**Rank sum**
0-20%	531	171278.5
21-40%	95	30951.5
>40%	17	4816
chi2(2) = 0.783		chi2(2) with ties = 0.783
Prob = 0.6762		Prob = 0.6761

EXH

**HRS_Clinic**	**Obs**	**Rank sum**
0-20%	531	171270
21-40%	95	30943.5
>40%	17	4832.5
chi2(2) = 0.744		chi2(2) with ties = 0.746
Prob = 0.6893		Prob = 0.6886

CYN

**HRS_Clinic**	**Obs**	**Rank sum**
0-20%	531	171392
21-40%	95	30756
>40%	17	4898
chi2(2) = 0.583		chi2(2) with ties = 0.584
Prob = 0.7471		Prob = 0.7467

PEFIC

**HRS_Clinic**	**Obs**	**Rank sum**
0-20%	531	168315
21-40%	95	31723.5
>40%	17	7007.5
chi2(2) = 4.789		chi2(2) with ties = 4.804
Prob = 0.0912		Prob = 0.0905

h. **Kruskal Wallis test to determine if total burnout scores (BO), exhaustion (EXH), cynicism (CYN), and professional efficacy (PEFIC) subscale of Maslach Burnout Inventory General Survey (MBI-GS) differs in number of committees (3 categories)**

Number of Committees:

BO

CommitCat	Obs	Rank sum
2 or fewer	45	14871.5
3 to 5	70	21424
6+	63	22010
None	467	150029.5
chi2(3) = 1.953		chi2(3) with ties = 1.954
Prob = 0.5822		Prob = 0.5821

EXH

CommitCat	Obs	Rank sum
2 or fewer	45	14593
3 to 5	70	21291.5
6+	63	21141.5
None	467	151309
chi2(3) = 1.018		chi2(3) with ties = 1.021
Prob = 0.7969		Prob = 0.7963

CYN

CommitCat	Obs	Rank sum
2 or fewer	45	15118.5
3 to 5	70	21753.5
6+	63	22635
None	467	148828
chi2(3) = 3.159		chi2(3) with ties = 3.165
Prob = 0.3678		Prob = 0.3669

PEFIC

CommitCat	Obs	Rank sum
2 or fewer	45	13872
3 to 5	70	23715.5
6+	63	20254
None	467	150493.5
chi2(3) = 0.796		chi2(3) with ties = 0.798
Prob = 0.8505		Prob = 0.8499

i. **Kruskal Wallis test to determine if total burnout scores (BO), exhaustion (EXH), cynicism (CYN), and professional efficacy (PEFIC) subscale of Maslach Burnout Inventory General Survey (MBI-GS) differs number of course taught (4 categories)**

Number of Courses Taught:

BO

Courses	Obs	Rank sum
1-2 courses	95	23845.5
3-4 courses	262	83024.5
5-6 courses	173	61897
6+ courses	114	38923
chi2(3) = 21.670		chi2(3) with ties = 21.678
Prob = 0.0001		Prob = 0.0001

EXH

Courses	Obs	Rank sum
1-2 courses	95	24935
3-4 courses	262	82129.5
5-6 courses	173	61592.5
6+ courses	114	39033
chi2(3) = 17.427		chi2(3) with ties = 17.472
Prob = 0.0006		Prob = 0.0006

CYN

Courses	Obs	Rank sum
1-2 courses	95	23549.5
3-4 courses	262	84326.5
5-6 courses	173	61026
6+ courses	114	38788
chi2(3) = 20.892		chi2(3) with ties = 20.932
Prob = 0.0001		Prob = 0.0001

PEFIC

Courses	Obs	Rank sum
1-2 courses	95	35593
3-4 courses	262	81696
5-6 courses	173	51888.5
6+ courses	114	38512.5
chi2(3) = 11.651		chi2(3) with ties = 11.687
Prob = 0.0087		Prob = 0.0085

Number of Courses (Dunn's Test):

Dunn's Pairwise Comparison of BO by Courses
(Bonferroni)

Col Mean-<br>Row Mean	1-2 cour	3-4 cour	5-6 cour
3-4 cour	-2.957319<br>0.0093		
5-6 cour	-4.495344<br>0.0000	-2.244352<br>0.0744	
6+ cours	-3.499287<br>0.0014	-1.175922<br>0.7189	0.728906<br>1.0000

Dunn's Pairwise Comparison of EXH by Courses
(Bonferroni)

Col Mean-<br>Row Mean	1-2 cour	3-4 cour	5-6 cour
3-4 cour	-2.291690<br>0.0658		
5-6 cour	-3.942748<br>0.0002	-2.337776<br>0.0582	
6+ cours	-3.096202<br>0.0059	-1.387346<br>0.4960	0.608132<br>1.0000

Dunn's Pairwise Comparison of CYN by Courses
(Bonferroni)

Col Mean-<br>Row Mean	1-2 cour	3-4 cour	5-6 cour
3-4 cour	-3.322779<br>0.0027		
5-6 cour	-4.417924<br>0.0000	-1.696657<br>0.2693	
6+ cours	-3.576758<br>0.0010	-0.881747<br>1.0000	0.557737<br>1.0000

Dunn's Pairwise Comparison of PEFIC by Courses
(Bonferroni)

Col Mean-<br>Row Mean	1-2 cour	3-4 cour	5-6 cour
3-4 cour	2.824909<br>0.0142		
5-6 cour	3.150332<br>0.0049	0.652997<br>1.0000	
6+ cours	1.427376<br>0.4604	-1.248048<br>0.6360	-1.691092<br>0.2725

j. Kruskal Wallis test to determine if total burnout scores (BO), exhaustion (EXH), cynicism (CYN), and professional efficacy (PEFIC) subscale of Maslach Burnout Inventory General Survey (MBI-GS) differs years worked (4 categories)

Years Worked:

BO

YrWorkedCat	Obs	Rank sum
1 year or less	45	12290.5
10+ years	264	86378.5
2-5 years	200	66033.5
6-10 years	136	43632.5
chi2(3) = 3.672		chi2(3) with ties = 3.674
Prob = 0.2991		Prob = 0.2989

EXH

YrWorkedCat	Obs	Rank sum
1 year or less	45	12405.5
10+ years	264	84021.5
2-5 years	200	67750.5
6-10 years	136	44157.5
chi2(3) = 4.513		chi2(3) with ties = 4.525
Prob = 0.2111		Prob = 0.2101

CYN

YrWorkedCat	Obs	Rank sum
1 year or less	45	12027.5
10+ years	264	88716
2-5 years	200	64146
6-10 years	136	43445.5
chi2(3) = 5.397		chi2(3) with ties = 5.407
Prob = 0.1449		Prob = 0.1443

PEFIC

YrWorkedCat	Obs	Rank sum
1 year or less	45	13275.5
10+ years	264	90614
2-5 years	200	60014.5
6-10 years	136	44431
chi2(3) = 7.210		chi2(3) with ties = 7.232
Prob = 0.0655		Prob = 0.0649

k. Kruskal Wallis test to determine if total burnout scores (BO), exhaustion (EXH), cynicism (CYN), and professional efficacy (PEFIC) subscale of Maslach Burnout Inventory General Survey (MBI-GS) differs in position (4 categories)

Position:

BO

PositionCat	Obs	Rank sum
Full time Permanent	453	151703.5
Full time Temporary	75	25215.5
Other	54	16298.5
Part-time	63	15117.5
chi2(3) = 15.429		chi2(3) with ties = 15.435
Prob = 0.0015		Prob = 0.0015

EXH

PositionCat	Obs	Rank sum
Full time Permanent	453	151518.5
Full time Temporary	75	26146
Other	54	15103
Part-time	63	15567.5
chi2(3) = 16.505		chi2(3) with ties = 16.548
Prob = 0.0009		Prob = 0.0009

CYN

PositionCat	Obs	Rank sum
Full time Permanent	453	151568
Full time Temporary	75	24297
Other	54	17268.5
Part-time	63	15201.5
chi2(3) = 13.882		chi2(3) with ties = 13.909
Prob = 0.0031		Prob = 0.003

PEFIC

PositionCat	Obs	Rank sum
Full time Permanent	453	146798.5
Full time Temporary	75	23466.5
Other	54	17638.5
Part-time	63	20431.5
chi2(3) = 0.259		chi2(3) with ties = 0.260
Prob = 0.9675		Prob = 0.9674

Position (Dunn's Test):

Dunn's Pairwise Comparison of BO by PositionCat
(Bonferroni)

Col Mean-<br>Row Mean	Full-tim	Full-tim	Other
Full-tim	-0.056850<br>1.0000		
Other	1.232687<br>0.6531	1.034070<br>0.9033	
Part-tim	3.789293<br>0.0005	3.022902<br>0.0075	1.790561<br>0.2201

Dunn's Pairwise Comparison of EXH by PositionCat
(Bonferroni)

Col Mean-<br>Row Mean	Full-tim	Full-tim	Other
Full-tim	-0.609289<br>1.0000		
Other	2.045113<br>0.1225	2.075303<br>0.1139	
Part-tim	3.491666<br>0.0014	3.191708<br>0.0042	0.944070<br>1.0000

Dunn's Pairwise Comparison of CYN by PositionCat
(Bonferroni)

Col Mean-<br>Row Mean	Full-tim	Full-tim	Other
Full-tim	0.457920<br>1.0000		
Other	0.552226<br>1.0000	0.125599<br>1.0000	
Part-tim	3.726959<br>0.0006	2.598356<br>0.0281	2.273609<br>0.0690
